# Probucol Prevents Diabetes-Induced Retinal Neuronal Degeneration through Upregulating Nrf2

**DOI:** 10.1155/2020/3862509

**Published:** 2020-02-13

**Authors:** Heng-Wei Liu, Yong Luo, Yu-Fan Zhou, Zhong-Ping Chen

**Affiliations:** ^1^Aier School of Ophthalmology, Central South University, Aier Eye Institute, Changsha 410015, Hunan Province, China; ^2^Department of Ophthalmology, Aier Eye Hospital of Changsha, Changsha 410015, Hunan Province, China

## Abstract

Diabetic retinopathy (DR) is a sight-threatening complication of diabetes. This study investigated the therapeutic effect of probucol in a mouse model of diabetic retinopathy. C57BL/6 mice were rendered diabetic through Streptozotocin (STZ) intraperitoneal injection. Mice were treated with probucol (150 mg/kg, gavage administration) or vehicle (DMSO) for 12 weeks. Optical coherence tomography (OCT), fundus photography (FP), and fundus fluorescein angiography (FFA) were conducted to evaluate retinal structure and damage. Eyes were collected for histology, reactive oxygen species (ROS) assay, apoptotic cells count, and western blot. After STZ injection, all mice developed hyperglycemia. Compared with the retina of the control group, the retina of diabetic mice showed enhanced arterial reflex and beaded vein dilatation. Besides, reduced inner and middle retinal thickness and significantly fewer nuclei were found in diabetic retina. Moreover, the diabetic retina also presented increased ROS generation and more TUNEL-positive cells. Probucol treatment prevented diabetes-induced lesions. In addition, the treatment also upregulated Nrf2 expression in diabetic retina. It was suggested that probucol attenuated diabetes-induced retinal neuronal degeneration via upregulating the Nrf2 signaling pathway possibly. Probucol may be repurposed for DR management.

## 1. Introduction

Diabetic retinopathy (DR) is the most common microvascular complication in diabetic patients and one of the major causes of acquired blindness worldwide [1]. The pathogenesis of DR is multifaceted and involves oxidative stress [2], proinflammatory changes [3], and the production of advanced glycation end products [4]. These processes lead to dysfunction of multiple types of retinal cells, including vascular endothelial cells, pericytes, neurons damages, and glial cells.

Preventative treatments for DR are limited, and most of the currently available treatments targeting DR are invasive. There is a growing awareness of the importance of identifying protective pathways related to this condition. Oxidative stress is considered to be one of the main pathogeneses of DR [5]. The Nrf2 signaling pathway is considered to be part of the most important cellular pathway protecting against oxidative stress [6]. The Nrf2 signaling pathway regulates the expression of a large battery of endogenous protective genes involved in the cellular antioxidant defense systems [7]. These protective genes play an important role in enhancing organizational antioxidant ability, as well as exerting antitoxin, antitumor, anti-inflammatory, and antiapoptotic effects [8–10].

Probucol is a bisphenol compound that functions as a lipid-lowering drug with anti-inflammatory and antioxidant properties and is widely used to lower cholesterol and reduce atherosclerosis in the clinic [11, 12]. There are certain reports in the literature which indicate that probucol acts as a potent oxygen radical scavenger and thus can effectively prevent oxidative stress-induced tissue damage [13, 14]. Duan et al. [15] and Yang et al. [16] found that probucol can improve the occurrence and development of diabetic nephropathy through antioxidation and protect against diabetic nephropathy. Mori et al. [17] found that probucol may show beneficial effects on diabetic retinopathy by preventing or slowing the impairment of the retinal circulation in patients with diabetes mellitus. Zhou et al. [18] showed that probucol can reduce oxidative stress and inhibit neuronal apoptosis after spinal cord injury by activating the Nrf2 signaling pathway. Previously, we have shown that probucol could inhibit intracellular reactive oxygen species (ROS) generation, promote proliferation, and decrease apoptosis of Müller cells under high glucose condition [19].

In this study, we investigated the efficacy of probucol in the development of diabetic retinopathy using a mouse model of STZ-induced diabetes. We found that probucol significantly reduced diabetes-induced retinal neuronal degeneration. We further found that probucol can upregulate Nrf2 expression in diabetic retina.

## 2. Materials and Methods

### 2.1. Experimental Animals and Experimental Design

Eight-week-old C57BL/6 mice (SPF grade) weighing 18–20 g were purchased from the Department of Laboratory Animals of Central South University, Changsha, China. All mice were housed in a 12-h light/12-h dark cycle at 22–25°C with free access to standard diet and tap water. The design and experimental procedures of the study were approved by the Animal Care Committee of the Central South University and complied fully with the protocol outlined in the Guide for the Association of Research for Vision and Ophthalmology Statement for the Use of Animals in Ophthalmic and Vision Research.

Diabetes was rendered via intraperitoneal injection of Streptozotocin (STZ: Sigma-Aldrich; Merck KGaA, Darmstadt, Germany) in citrate buffer (pH 4.5) of a single dose of 150 mg/kg. The nondiabetic control mice were injected with citrate buffer only. Nonfasting blood glucose level was measured using blood drawn from the tail vein one week after the injection of STZ. Blood glucose ≥16.7 mmol/L was considered as diabetic. Subsequently, diabetic mice were randomized into three groups: (1) the diabetes + probucol group (DM + PB); (2) the diabetes + vehicle (DMSO) group (DM + DMSO); and the (3) diabetes nontreatment control group (DM). Drug intervention was started after the confirmation of hyperglycemia and lasted for 12 weeks. The DM + PB group were administrated with probucol (150 mg/kg in 1% DMSO, Shandong Qilu Pharmaceutical Co., Shandong, China) via daily oral gavaging. The DM + DMSO group received 1% DMSO via daily oral gavaging. Body weight and nonfasting blood glucose levels were monitored weekly. At the end of the studies, mice were sacrificed by an overdose of pentobarbitone sodium injection, and eyes were collected for further investigations.

### 2.2. In Vivo Retinal Imaging

Retinal morphological changes were monitored in vivo using fundus photography, fundus fluorescein angiography (FFA), and optic coherence tomography (OCT) using the micron IV system (Phoenix Research Labs, USA) at different times (weeks 0, 4, 8, and 12 after drug administration). Mice were anesthetized via an intraperitoneal injection of pentobarbitone sodium (35 mg/kg), and the eyes were dilated with 1% tropicamide. The medical sodium hyaluronate gel was used to prevent corneal dryness. After color fundus photography, sodium fluorescein (10%; 0.05 mL) was injected intraperitoneally, and FFA images were collected immediately. OCT images were then collected using a high-definition circular scan mode. The retinal thickness of the eyes was measured through the OCT module. Three retinal layers including the inner layer (from the retinal nerve fiber layer (NFL) to the inner plexiform layer (IPL)), the middle layer (from the inner nuclear layer (INL) to the inner and outer segments (IS/OS) of the photoreceptors), and the outer layer (the end of inner and outer segments (IS/OS) of the photoreceptors to the retinal pigment epithelium (RPE)) were segmented using the Insight software (Phoenix Research Labs, USA), and the thickness of each layer was measured.

### 2.3. Ocular Histology

Eyes were collected at the end of the study and fixed in 4% paraformaldehyde for two hours before being processed in an automated tissue processor (Wuhan Junjie Electronic Co., Ltd.). The eyes were embedded in paraffin, and radial 5-*μ*m sections were achieved using a microtome (Leica RM2016, Heidelberg, Germany). Standard hematoxylin and eosin (H&E) staining was performed. The number of cell nuclei in the inner nuclear layer (INL) and the outer nuclear layer (ONL) were counted using ImageJ software.

### 2.4. ROS Assay

Eyes were collected at the end of the study and embedded in OCT (Tissue-Tek O.C.T. Compound 4583, USA). The OCT specimens were immersed in liquid nitrogen and then cryosectioned into 10 microns thickness. Unfixed frozen cross sections were incubated with dihydroethidium (DHE) (5 mM; TargetMol, USA) in a light-protected moist chamber at 37°C for 20 min. Images were obtained with a fluorescence microscope using the same imaging settings for each sample. For semiquantitative analysis of ROS production, total fluorescence intensity was analysed with the ImageJ software using four images from sections per eye for each experimental condition.

### 2.5. TUNEL Assay

Apoptotic cells were detected using the TUNEL (Terminal deoxynucleotidyl transferase-mediated dUTP Nick End-Labeling) assay on paraffin-embedded sections using the In Situ Cell Death Detection Kit (POD, Sigma-Aldrich) according to the manufacturer's instructions. Images were acquired using a fluorescence microscope. We counted the TUNEL-positive cells of the ganglion cell layer (GCL) and the entire retinal layer.

### 2.6. Western Blot

The eyes were collected and retinas were dissected and homogenized in RIPA buffer containing phenylmethane sulfonyl fluoride (PMSF, Beyotime Biotechnology, Nanjing, China). The retinal specimen was further homogenized with ultrasound at 4°C for 3–5 sec and incubated for 30 min. The retinal lysis was centrifuged at 14000 rpm for 10 min at 4°C. The supernatant was collected, and protein concentration was determined by BCA protein concentration detection. Approximately 15 *μ*g of total protein was separated by sodium dodecyl sulfate-polyacrylamide gel electrophoresis (SDS-PAGE). Subsequently, the separated proteins were transferred onto a polyvinylidene uoride (PVDF) membrane and blocked with 5% skim milk powder in TBST (25 mM Tris-HCl, 0.15 M saline, and 1% Tween 20) at room temperature for 1 h. Membranes were then incubated overnight at 4°C with the primary antibodies : Nrf2 (1 : 500; Abcam, UK) or GAPDH (1 : 1000; Boster,China). After washing the membranes three times with TBST (5 min per wash), the membranes were incubated with the secondary antibody which was combined with horse radish peroxidase (HRP). Immunoreactive bands were visualized by ImageQuant LAS 500 (GE Healthcare, USA) and quantified by greyscale analysis by using ImageJ software.

### 2.7. Statistical Analysis

GraphPad Prism (version 8) was used to analyze data, and data are presented as mean ± SEM. The differences between groups were analyzed by one-way ANOVA followed by Bonferroni's multiple comparisons test. A *P* value less than 0.05 was considered statistically significant.

## 3. Results

### 3.1. Probucol Treatment Did Not Alter Blood Glucose Levels

Body weight and nonfasting blood glucose levels were monitored routinely. STZ intraperitoneal injection successfully induced hyperglycemia (see Table 1). Daily administration of probucol did not change the nonfasting blood glucose levels.

### 3.2. Probucol Attenuated Diabetic-Induced Retinal Neuronal Degeneration

**Figure 1:**
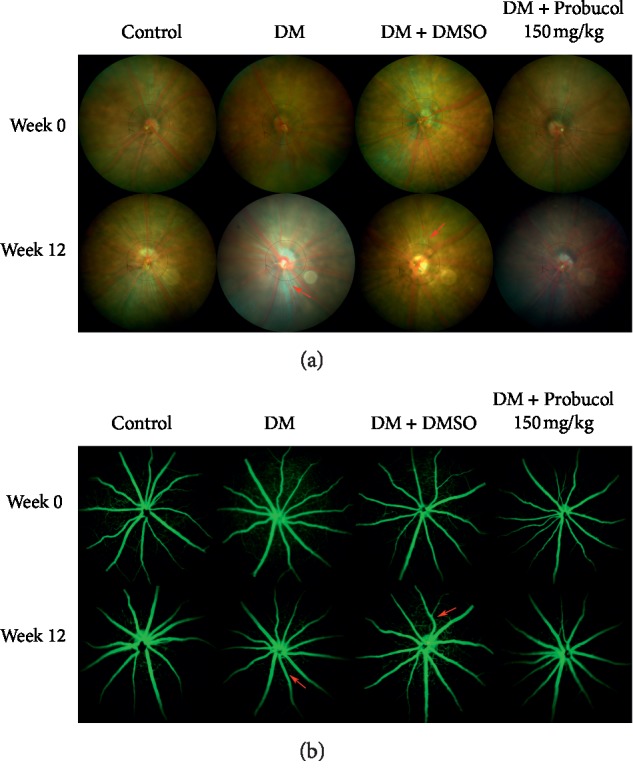
Ocular fundus images and fluorescein angiography. (a) Representative fundus images at week 0 and week 12. (b) Representative fluorescein angiography at week 0 and week 12.

Fundus imaging and fluorescent angiography were performed to observe the possible alternation of fundus or blood vessel structures at week 0 (before the administration of probucol), weeks 4, 8, and 12 (after probucol treatment in STZ-injected mice).  Figure 1 shows the representative images of all experimental groups at week 0 and week 12. In healthy retinas, the FP and FFA results show a clear region of full fundi and fluorescent images. At week 12, the fundi of the DM and DM + DMSO group were observed to have enhanced arterial reflex and beaded vein dilatation (red arrows), although there was no visible evidence of microvascular abnormalities.

**Figure 2:**
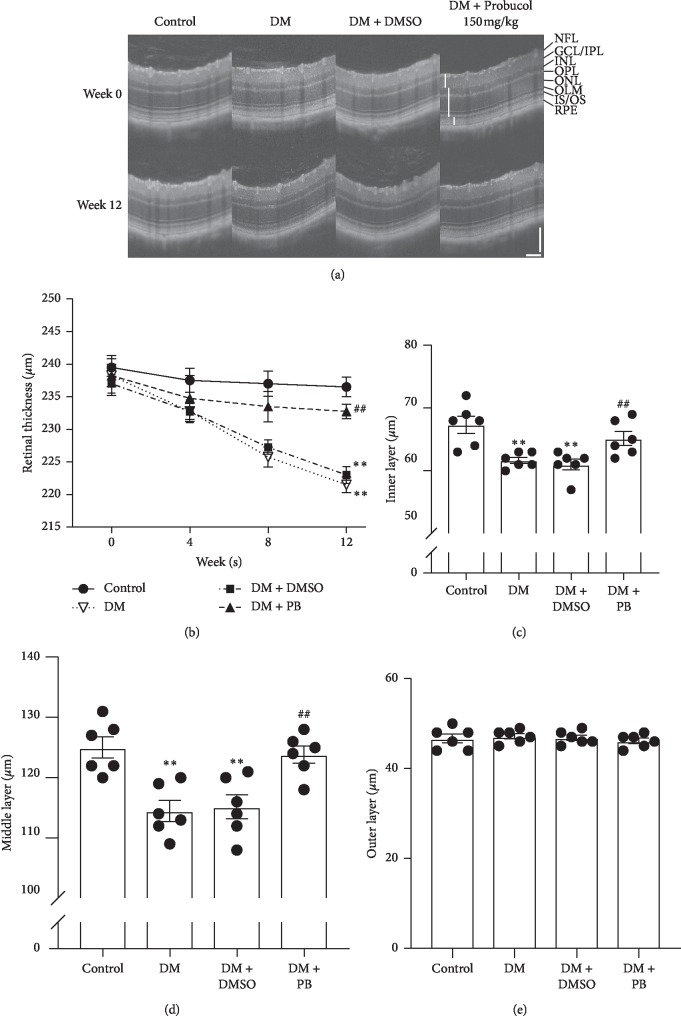
Spectral domain optical coherence tomography (SD-OCT) examination revealed in vivo retinal alterations in C57BL/6 mice. (a) Representative SD-OCT images at week 0 and week 12. A definition of the retinal layers is shown on the right-hand side of the panel. (b) Retinal total thickness obtained at 4-week intervals from SD-OCT in the control group, DM group, DM + DMSO group, and DM + PB group. (c–e) Retinal thickness of each layer at week 12 measured using SD-OCT. (c) Inner retinal layer: from NFL to IPL; (d) middle retinal layer: from INL to IS/OS; and (e) outer retinal layer: from IS/OS to RPE. Mean ± SEM, *N* = 6 mice per group. ^*∗∗*^*p* < 0.01 compared with the control group. ##*p* < 0.01 compared with the untreated DM group. Scale bar: 100 *μ*m.

OCT was used to monitor the retinal structure at week 0, 4, 8, and 12.  Figure 2(a) shows the representative images of all experimental groups at week 0 and week 12. Detailed retinal layers were observed in all groups, and no obvious dysmorphology was observed in any of the diabetes groups. However, the total retinal thickness was reduced in DM and DM + DMSO groups in a time-dependent manner (see Figure 2(b)). Further analysis of different retinal layers showed that retinal neuronal degeneration occurred mainly in the inner layer and middle layer. Probucol treatment significantly attenuated diabetes-induced reduction of retinal thickness in both inner and middle layers (see Figures 2(c), 2(d)). The thickness of the outer retinal layer was not affected in diabetic mice (see Figure 2(e)).

### 3.3. Effects of Probucol on Ocular Histological Damage and Retina Oxidative Injury in Diabetic Mice

**Figure 3:**
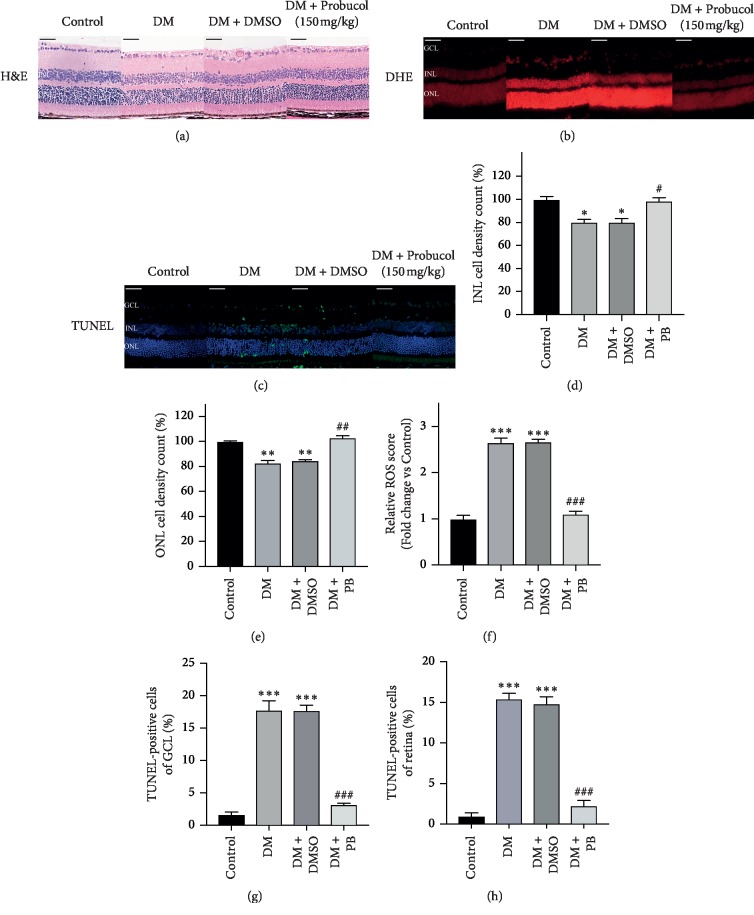
Ocular histological and oxidative injury analysis examination of the effects of probucol on streptozotocin- (STZ-) induced diabetic retinopathy in C57BL/6 mice. (a) Representative images of H&E staining showing the morphology of the eyes from the control group, DM group, DM + DMSO group, and DM + PB group at week 12. (b) Representative images of Dihydroethidium (DHE) staining in retinas in each group. (c) Representative images of TUNEL staining in retinas in each group.(d-e) Nuclei number counting of the inner nuclear layer and outer nuclear layer demonstrated that there are less nuclei in the DM and DM + DMSO groups. (f) Bar graphs representing quantification of tissues stained with DHE. (g-h) Bar graphs representing quantification of tissues stained with TUNEL demonstrated that there are more TUNEL-positive cells in the DM and DM + DMSO groups. Mean ± SEM, *N* = 4 mice per group. ^*∗*^*p* < 0.05; ^*∗∗*^*p* < 0.01; ^*∗∗∗*^*p* < 0.001 compared with the control group; #*p* < 0.05, ##*p* < 0.01; ###*p* < 0.001 compared with the DM group. Scale bar: 50 *μ*m.

To determine the effects of probucol on ocular histological damage, H&E staining revealed significantly structural disturbances and cell loss in DM and DM + DMSO groups (see Figure 3(a)). The nuclear number in the INL and ONL of each condition was counted. The total number of nuclei of the INL and ONL was significantly lower in DM and DM + DMSO groups (see Figures 3(d), 3(e)). Reactive oxygen species (ROS) generation was increased in the retina of DM and DM + DMSO groups, which is shown by staining with DHE, an indicator of oxidation (see Figure 3(b)). Probucol administration significantly decreased DHE staining in STZ-induced diabetic mice (see Figure 3(b)). No significant differences in DHE staining were noted between DM and DM + DMSO groups (see Figure 3(f)). To characterize cell loss observed in the retina, we used the terminal deoxynucleotidyl transferase-mediated dUTP nick end-labeling (TUNEL) assay. The retina of DM and DM + DMSO groups showed slight TUNEL-positive cells (Green fluorescence), whereas the retina from control and DM + PB groups had almost no TUNEL-positive cells (see Figure 3(c)). The results further confirmed that retinal neurondegeneration occurred in diabetic retina. Probucol treatment prevented the neurondegeneration which may be related to its ability to suppress ROS generation.

### 3.4. Probucol Treatment Upregulated Nrf2 in Diabetic Retina

**Figure 4:**
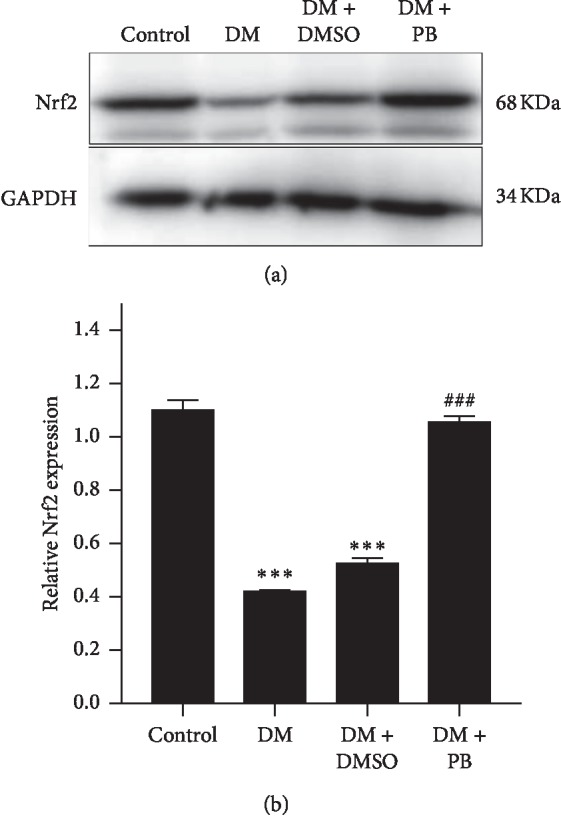
Nrf2 expression in the retina from different groups. Proteins were extracted from retinal tissue, and western blot was used to detect Nrf2 expression. (a) Representative western blotting showing Nrf2 expression and housekeeping protein GAPDH in all experimental conditions. (b) Bar graph showing quantification of Nrf2 expression in western blot. Intensity of Nrf2 expression was normalized to that of GAPDH. Mean ± SEM, *N* = 4 mice per group, ^*∗∗∗*^*p* < 0.001 compared with the control group; ###*p* < 0.001 compared with the DM group.

To explore the potential mechanism of probucol-mediated protection on diabetic retina, we performed Western blot of Nrf2 on retinal tissues. Nrf2 expression levels were significantly reduced in DM and DM + DMSO groups (see Figure 4), probucol significantly increased the expression of Nrf2 in diabetic retina. The difference between DM groups and the DM + DMSO groups had no statistical significance.

## 4. Discussion

Diabetic retinopathy is a combination of retinal neuropathy and vasculopathy [20–22]. In this study, using a STZ-induced mouse model, we found that probucol attenuated the diabetes-induced neuropathy and vasculopathy via upregulating the Nrf2. To our knowledge, this is the first animal study to investigate the protective effect of probucol on diabetic retinopathy through the Nrf2 pathway.

Oxidative stress is considered the main pathogenesis of DR [5]. The balance between mitochondrial ROS or oxygen-free radical levels and the antioxidant-reduction defense system in retinal cells of diabetic patients is disrupted: In hyperglycemic environment, ROS in mitochondria increases, leading to imbalance of cellular redox system, and oxidative stress occurs. ROS is a free radical with impaired electron that usually participates in the redox mechanisms of some molecules such as enzymes, proteins, and so on. There are many efficient antioxidant defense systems in the body, including the antioxidant defense enzymes. Once the antioxidant system is compromised, elimination of free radicals becomes impaired. Excess oxygen free radicals can connect with proteins and cross-link with DNA, leading to abnormal reactions that may severely interfere with normal metabolisms, induce cell apoptosis and neovascularization in the retina, and ultimately promote the development of DR [23].

Probucol is a lipid-lowering drug which increases the fraction rate of LDL catabolism and has been used to treat hyperlipidemia [24]. The antioxidant and anti-inflammatory properties of probucol have been investigated using in vitro cell cultures and in vivo animal models [13, 14]. It inhibits extracellular ROS and effectively reduces vascular endothelial cell apoptosis caused by oxidative stress damage [25]. Probucol reduces hypoxia-induced angiogenesis by improving the function of endothelial progenitor cells [26]. Previous studies also found that probucol inhibited ventricular remodeling and improved diabetic nephropathy in diabetic rats through antioxidation [15]. A previous report by our group [19] showed that probucol can inhibit intracellular ROS generation, promote proliferation, and decrease apoptosis of Müller cells under high glucose conditions. In this study, we found that probucol not only attenuates diabetic-induced retinal neuronal degeneration but also inhibits ROS generation in a STZ-induced type 1 diabetic model. We speculate that probucol may attenuate the development of DR through its antioxidant effect.

Nuclear factor erythroid-derived factor 2-related factor 2(Nrf2) is expressed in all tissues ubiquitously. It serves as a transcription factor that protects cells from endogenous and exogenous stresses [27]. The Nrf2 molecule binds to an antioxidant response element (ARE), thereby regulating the expression of a large battery of cytoprotective genes involved in the cellular antioxidant responses. Under normal conditions, Nrf2 is sequestered in the cytoplasm by Kelch-like ECH-associated protein 1 (Keap1). Under conditions of oxidative stress, Keap 1 releases Nrf2, which is translocated to the nucleus where it activates an ARE [28, 29]. The Nrf2 signaling pathway regulates more than 200 endogenous protective genes involved in the cellular antioxidant defense systems [29]. The Nrf2 signaling pathway is considered to be part of the most important cellular pathway protecting against oxidative stress [30]. Studies have shown that inhibition of Nrf2 significantly reduces the expression of antioxidant genes and exacerbates oxidative stress levels in human and mouse retinas, and Nrf2 knockout mice exhibit early DR blood retinal barrier dysfunction [31, 32]. Through the study of the DM rat model, it is found that regulation of Nrf2 by drug or molecular level can inhibit oxidative stress and inhibit the development of DR [33]. The conspicuous impairment of Nrf2 activation contributes to the severity of oxidative stress, inflammation, and the progression of tissue damage in the kidney [34]; thus, the therapeutic activation of Nrf2 could be used as a strategy to prevent or slow the progression of diabetic nephropathy [35]. Furthermore, decreasing the expression of Nrf2 exacerbated ROS production in endothelial cells in both normoglycemic and hyperglycemic conditions [36]. This suggests the prevention of intracellular ROS formation might be largely dependent on Nrf2. The activation of Nrf2 via pharmacological intervention is now considered to be an effective strategy for the prevention of diabetic complications. In our study, Nrf2 expression level was significantly reduced in diabetic retina, while probucol increased Nrf2 expression and reduced ROS production, suggesting that probucol may exert an antioxidant effect via upregulating the Nrf2 pathway. However, the underlying mechanism of this process requires further research.

## 5. Conclusion

We found that probucol attenuated diabetes-induced retinal neuronal degeneration. Probucol may be repurposed for the management of DR and other diabetes-induced complications.

## Figures and Tables

**Table 1:** Body weight and blood glucose levels in C57BL/6 mice.

Groups	Body weight, g	Blood glucose, mmol/L
Control	28.71 ± 0.33	7.55 ± 1.48
DM	20.73 ± 0.84^*∗∗∗*^	27.90 ± 0.33^*∗∗∗*^
DM + DMSO	21.58 ± 0.81^*∗∗∗*^	26.62 ± 1.28^*∗∗∗*^
DM + PB	21.22 ± 0.87^*∗∗∗*^	26.67 ± 1.18^*∗∗∗*^

^*∗∗∗*^ *p* < 0.001 vs. the control group. *N* = 6 mice per group. Data are the mean ± SEM.

## Data Availability

The data used to support the findings of this study are included within the article.
